# The Influence of Phyto-Active Incorporated Topical Formulations on Cell Migration in Scratch Assays and *In vivo* Wound Model in Mice

**DOI:** 10.2174/0115672018381444251009112958

**Published:** 2026-01-08

**Authors:** Evren Algın Yapar, Evren Homan Gökçe, Ebrar İnal, Şeyma Ulusoy, İffet İrem Tatlı Çankaya, Murat Kartal

**Affiliations:** 1 Department of Pharmaceutical Technology, Faculty of Pharmacy, Sivas Cumhuriyet University, Sivas, Türkiye;; 2 Department of Pharmaceutical Technology, Faculty of Pharmacy, Ege University, İzmir, Türkiye;; 3 Department of Pharmacognosy, Faculty of Pharmacy, Bezmialem Vakıf University, Istanbul, Türkiye;; 4 Institute of Health Sciences, Bezmialem Vakıf University, Department of Pharmacognosy and Natural Product Chemistry, Istanbul, Türkiye;; 5 Department of Pharmaceutical Botany, Faculty of Pharmacy, Hacettepe University, Ankara, Türkiye;; 6 Phytotheraphy Research and Application Center, Bezmialem Vakıf University, Istanbul, Türkiye

**Keywords:** Phytomedicine, wound healing, rheology, texture analysis, scratch assay, *in vivo* burn model, histological examinations

## Abstract

**Introduction:**

*Vitis vinifera* L. seed oil, *Trigonella foenum-graecum* L. seed oil, and the *Olea europaea* L. oil macerates of *Helichrysum italicum* (Roth) G. flowers and *Matricaria recutita* L. flowers were used for the preparation of topical wound-healing ointments.

**Methods:**

The ointments basically were prepared by hot-melt blending method and subjected to rheological tests and texture profile analysis. After characterization of *in vitro* characterization studies, a scratch assay was conducted to evaluate the efficacy of ointment formulations. Ultimately, the optimized formulations underwent further testing on an *in vivo* burn wound model in mice.

**Results:**

Measured viscosity values were F1:382.98 Pa.s and F2:2562.3 Pa.s, respectively, and both of the formulations created an easy-to-apply, soft, thin adhesive film layer. The fast wound closure was observed with F1 formulation, and when applied at different doses of 100 µL, 200 µL, and 400 µL, the 200 µL concentration of F1 formulation was able to heal the wound totally (100%) at 48^th^ hour.

**Discussion:**

The F1 formulation presented lower viscosity than the F2; the increase in the white petrolatum concentration increased the initial viscosity as expected. F1 formulation had higher phyto-actives and *cera alba* and lower petrolatum in comparison to F2. The wound healing effects of both the formulations were synergistic due to their phytoactives content. In *in vivo* studies, the F1 ointment exhibited faster re-epithelialization with less inflammation compared to the burn control group.

**Conclusion:**

The best formulation included oils of *H. italicum, M. recutita, V. vinifera*, and *T. foenum-graecum* at a total concentration of 16%, exhibiting appropriate preadability and successful healing property. Additional research needs to be carried out to shed light on the mechanism underlying the formulation's healing capabilities.

## 1 INTRODUCTION

In recent years, the integration of medicinal and aromatic plants, long utilized in traditional therapies, into modern healthcare has gained considerable prominence. This strategy builds on the extensive historical use of these herbal resources, which have demonstrated efficacy and safety over centuries of human application. By employing these well-established botanical sources, the time and financial costs associated with developing novel synthetic compounds can be mitigated. Furthermore, these plants present a rational advantage due to their multifunctional therapeutic properties, arising from a diverse array of phytochemicals. The biological activity of these phytochemicals is attributable to the presence of various chemical classes. Among these, terpenes and terpenoids have emerged as promising agents in the prevention and treatment of diseases, including inflammatory disorders, tumorigenesis, and neurodegenerative conditions, as demonstrated in numerous cell and animal model studies over several decades. Additionally, polyphenols, including tannins, flavonoids, and lignin-carbohydrate complexes, are renowned for their potent antimicrobial, anti-inflammatory, and antioxidant properties [1]. A range of medicinal plants, such as goldenrod [*Helichrysum italicum* (Roth) G.], chamomile (*Matricaria recutita* L.), fenugreek (*Trigonella foenum-graecum* L.), grape (*Vitis vinifera* L.), and olive (*Olea europaea* L. subsp. *europaea*), have found utility in both traditional and conventional medicine. These plants are either naturally occurring or cultivated in Türkiye. *Helichrysum italicum* (golden herb), with high economic value and primarily distributed across Europe, is traditionally employed for wounds, burns, and scars, with olive oil macerate applications extended to cosmetics [2]. *Matricaria recutita* L. (chamomile), well-known in cosmetic applications, is evidenced to possess wound healing and anti-inflammatory properties when used as an olive oil macerate of its flowers [3]. The seed oils of *Vitis vinifera* L. (grape) and *Trigonella foenum-graecum* L. (fenugreek) further exhibit antioxidant properties that support wound healing [4, 5], and olive (*Olea europaea* L. subsp. *europaea*) fruit oil is extensively used in medicinal and cosmetic formulations, including wound healing ointments [6]. The European Union's Cosmetics Regulation (EC No. 1223/2009 Annexes), which delineates the legal status of such ingredients based on European Commission Consumer Safety reports, places no restrictions on the external use of these botanical components, thereby supporting their integration into topical formulations [7]. Numerous cosmeceuticals and non-prescription products utilize diverse combinations of herbal ingredients, particularly in skincare, wound, and burn ointments or creams, with extensive scientific studies corroborating their therapeutic potential [8]. Below stated studies have briefly explained the activity of the above-mentioned herbal sources in wound healing.

*V. vinifera* seeds increased TGF-β1 and VEGF expressions, thus accelerating wound healing [9]. In a study conducted on this subject, when the gene secretion of wound tissues was investigated, an elevation in TGF-β1 expression was observed in wound tissues treated with *V. vinifera* [4]. The relative protein secretion of VEGF was enhanced in wound tissues treated with *V. vinifera* seeds compared to untreated wound tissues. In addition, type I collagen in skin tissue plays an important role in healing connective tissue. The role of collagen in wound healing is to transport fibroblasts to the wound bed. Chronic wounds produce MMPs, and collagen stops the production of excess MMPs. Therefore, the upregulation of the relative secretion of type I collagen by *V. vinifera* seeds can prevent long-term inflammation and thus promote wound healing. Although studies have been conducted on seed extract, GC/MS analysis has been performed for the seed oil content in the extract, which contributes to wound healing and has a synergistic effect. Palmitic, azelaic, and stearic acids were the most abundant SFA in *V. vinifera* seed oil. Among the unsaturated fatty acids (UFAs), 9-hexadecenoic, 9-octadecenoic, and cis-11-eicosenoic acids were the most abundant monounsaturated FAs (MUFA). Combined n-2, and n-3 polyunsaturated fatty acids (PUFA) (18:2, C18:2, and C18:3) contained 9,12-octadecadienoic acid, 12,15-octadecadienoic acid, and 6-cis,9-cis,11-trans-octadecatrienoic acid, as major ones [4, 10, 11]. Fibrin is an important element in the main stage of skin healing. It allows fibroblasts to gather at the wound site and stimulates collagen production [12], and when they reach the wound site, they initiate a prolonged inflammatory phase that leads to wound remodeling and closure [13]. From day 3 onwards, a proliferative phase is triggered, characterized by angiogenesis, fibroblast migration, and the formation of granulation tissue, including collagen synthesis [14]. In the study by Rekik *et al*., significant wound healing was observed in the treated groups by *Vitis vinifera* seed oil from day 3 onward, with improved re-epithelialization [15]. *V. vinifera* seed oil contains significant amounts of polyunsaturated fatty acids (mainly oleic, linoleic, and linolenic acids), and linoleic acid, the precursor of arachidonic acid, is very critical in the inflammation pathway (prostaglandins, thromboxanes, and leukotrienes) [16]. These phytocompounds accelerate the inflammatory process. Thus, they elevate local neovascularization, extracellular matrix remodeling, migration, and fibroblastic cell differentiation [14], accelerating wound healing. It has also been reported that fatty acids can decrease transepidermal water loss, increase skin hydration, and provide a supportive environment for accelerated skin wound healing [17]. Furthermore, the wound healing effect of *V. vinifera* seed oil can be associated with the synergism between its antibacterial and antioxidant effects [15, 18].

Recent studies have indicated fenugreek to have antioxidant and anti-inflammatory effects. When applied to a wound, it accelerates the healing process and reduces inflammation. At this point, it is the fatty acids in fenugreek seeds that support wound healing and maintain skin elasticity by forming collagen. In particular, polyunsaturated fatty acids, especially linoleic acid, are effective in wound healing and provide epidermal healing, *i.e*., reepithelialization, which refers to the renewal of skin damaged by wounds or burns [19-24]. In the study conducted by Ali *et al*., epithelialization was revealed to be faster in the group treated with fenugreek seeds and it increased over time [23]. This indicated rapid epithelialization and collagenation of the proliferation phase [25-27]. Phytochemical analysis of fenugreek seed oil obtained by cold pressing method using GC-MS demonstrated the existence of eugenol, dihydrocoumarin, heptadecanoic acid, tri- and tetradecanoic acid, and hexadecanoic acid. Histopathological evaluation of the study conducted using the *in vivo* experimental model showed minimal tissue infiltration with inflammatory cells in animals subjected to fenugreek seed oil. In addition, TNF-*α* level in serum was significantly down-regulated, while SOD and CAT levels were up-regulated. These results revealed the antiangiogenic activity of fenugreek seed oil [28].

Preparations containing plants with high anti-inflammatory capacity are quite useful in the wound healing process [29-31]. Of these, *M. chamomilla* was included in the formulation because of its high wound healing capacity obtained on both incision and excision animal models. Significantly elevated wound closure percentage, faster re-epithelialization, elevated wound-breaking strength, elevated hydroxyproline amount, and higher collagen fiber percentage were exhibited by chamomile-treated groups [32-36]. Clinical studies have also justified the use of *Matricaria* in wound healing formulations. Topical application of chamomile extract [37] showed significantly faster wound healing and epithelialization in patients undergoing tattoo dermabrasion compared to the controls. Maiche *et al*. stated the limited subsequent radiation-induced skin reactions in areas treated with chamomile [38]. Merfort *et al*. also reported that the bioactive compounds of chamomile, apigenin, luteolin, and apigenin 7-O-β-glucoside, can penetrate deeper skin layers and can be utilized successfully as topical antiphlogistic agents [39].

*Helichrysum* species, known as "Heleiochrysos" in Historia Plantarum (3rd-2nd century BC), can be used in the treatment of burns (mixed with honey) and stings and bites of poisonous animals [40]. It has also been reported that *Helichrysum italicum* is utilized in inflammatory and allergic conditions and skin disorders [41, 42]. *H. italicum* has gained significant recognition in recent years for its association with cell regeneration and anti-aging skincare. It is quite popular in natural skin care products and can reduce scars [43]. *Helichrysum* is known as the "liquid suture" due to its extraordinary healing properties on the skin surface. The essential oil of the plant accelerates cell growth and helps wound healing and skin tissue regeneration [44]. Battaglia also stated that *H. italicum* can be used in the treatment of allergies, eczema, rashes, and psoriasis [45]. *H. italicum* extract and its major component, arzanol, inhibited TNF-*α*-induced HIV-1-LTR transactivation [46]. The flavonoid fraction of *H. italicum* flowers was also applied to patients without exposure to UVB radiation to investigate their photoprotective and anti-erythematous effects. It was found that both the crude extract and the flavonoid fraction inhibited the onset of the erythematous response [47]. The proposed mechanism of action of the flavonoid fraction may include inhibition of local prostaglandin production in irradiated skin, particularly through the luteolin effect [48], and inhibition of histamine release and radical scavenging activity *via* apigenin [49, 50]. *H. italicum* flavonoids may be useful in the formulation of products for burn treatment, radioprotection, and sunscreen effect. The effects of flavonoids were studied against a chronic inflammation model induced by multiple applications of TPA, and the most effective compound was found to be tiliroside, the mechanism of action of which was stated as reducing edema formation and neutrophil infiltration [51]. The importance of *H. italicum* extracts and isolated components as anti-inflammatory and antimicrobial agents has been proven [52, 53].

*Olea europaea* L. has been proven to possess anti-inflammatory and antioxidant properties and is, therefore, widely used in traditional herbal preparations [54]. These effects are mainly due to the presence of oleuropein. In a study comparing oleuropein-enriched *O. europaea* extract with Madecassol® ointment, it showed significantly higher wound contraction percentage and significantly higher wound tensile strength in an animal model of incision [55]. Oleuropein accelerated wound healing, decreased cell infiltration in the wound zone, and caused a significant increase in type 1 collagen fiber deposition and faster repithelialization [56]. In a similar study conducted by Mehraein *et al*., oleuropein has been shown to have excellent wound healing potential by accelerating the re-epithelialization process, increasing collagen fiber production, and elevating blood flow to the wounded area through up-regulation of vascular endothelial growth factor protein secretion [57].

In addition, it should not be overlooked that beeswax in the formulation was used not only as an emulsifier and thickener, but also for wound isolation and protection against microbial infection [58, 59].

In a recent study, a commercially available herbal wound and burn cream comprising *Centella asiatica* (L.) Urban was employed as a reference. Topical herbal formulations of *Hypericum perforatum* L. olive oil macerate were evaluated for their diabetic wound healing effects in *in vivo* rat and mouse models [60]. Another notable traditional Chinese medicine ointment, "Jinchuang", widely used at China Medical University Hospital for treating diabetic foot ulcers and pressure sores, has been found to combine multiple herbal extracts, including camphor [*Cinnamomum camphora* (L.) J. Presl], frankincense (*Boswellia serrata* Roxb.), dragon's blood [*Daemonorops draco* (Willd.) Blume], myrrh (*Commiphora myrrha* L.), and catechu [*Acacia catechu* (L.f.) Willd.]. *In vitro* assays have demonstrated this formulation to promote wound healing by enhancing cell proliferation, migration, and angiogenesis [61]. Kampo herbal ointments from Japan for wound healing are also widespread in this region. Ointments have a common lipophilic base of sesame oil and beeswax and include drug crudes from the genus of *Angelica*, *Lithospermum*, *Curcuma*, *Phellodendron*, *Paeonia*, *Rheum*, *Rehmannia*, *Scrophularia*, or *Cinnamomum* [8]. *Calendula officinalis* Linn. extract and white wax combination is another researched formula for wound healing [62]. Preclinical and clinical studies have also shown preparations prepared as herbal mixtures to have a wound healing effect due to synergistic effects [58].

In a study conducted by Akhoondinasab *et al*., two traditional herbal ointments (Robacin: *Rosa × damascena* Herrm., *Calendula officinalis* L., and beeswax; Rimojen: *Thymus serpyllum* L., macrophyllum, and platonychium) and *Aloe vera* (L.) Burm.f. extract were compared with silver sulfadiazine used in standard treatment for burn wounds. Compared to silver sulfadiazine cream, a significant decrease in wound surface area was achieved in rats treated with Robacin, and the healing rate was also higher than in the other groups. In terms of wound surface area, maximum healing was observed simultaneously in second and third-degree burn wounds in the Robacin group, in second-degree burns in the *Aloe vera* and Rimojen groups, and third-degree burn wounds in the *Aloe vera* and silver sulfadiazine groups. Pathologically, epithelialization was more pronounced in both the scars of the *Aloe vera* group and third-degree burns in the Robacin group. Histological examination showed minimal angiogenesis and fibrosis rate in the Robacin group, indicating less scarring in this group. The possible mechanism involved in wound healing can be explained as increasing blood flow in the burn area, reducing inflammatory response, and reducing infection rate in the relevant area [63].

The healing effect of a traditional ointment consisting of olive oil, sheep fat, beeswax, turmeric, salt, henna, egg yolk, and natural gum on the healing of grade II burn wounds in mice was compared with an effective agent, silver sulfadiazine (SSD). The results were documented on 3 days (days 0, 7, and 14). Half of the mice were selected for histopathological and morphometric evaluation and wound size changes on day 7, and the rest for the same evaluations on day 14. Microscopic and macroscopic evaluations showed that the ointment had the same effect as the SSD cream in reducing inflammation and enhancing the healing process. There was no significant effect of this oinment or SSD on the size of the wounds during the experiment, indicating time to be the only factor affecting the size of the wound in all groups [64].

In a comparative study conducted using silver sulfadiazine (SS) and poly herbal complex (PHC) consisting of aqueous extracts of *Malva sylvestris* L. and *Solanum nigrum* L. leaves together with oil extract of *Rosa × damascena* petals, it was observed that although SS showed better wound healing effect in the first days of treatment, at the end of the treatment period, PHC had significant potential for burn wound healing compared to SS. Moreover, histopathological evaluation showed complete re-epithelialization, well-formed granulation tissue, and neovascularization, further strengthening the potential of PHC in burn wound healing. Antioxidant and antibacterial activities of PHC against *Staphylococcus aureus*, a common cause of skin and soft tissue infections, were also determined; at the end of the treatment period, a significant improvement in the healing percentage was observed in PHC-treated rats compared to the other groups. The wounds healed in PHC-treated animals contained less inflammatory cells and had desirable re-epithelialization with remarkable neovascularization. It also showed antioxidant activity and antibacterial activity against *S. aureus*. The activity of the cream was attributed to the phenolic and tannin phytochemicals it contained [65].

In another study conducted to compare the effectiveness of thymoquinone (TQ), a traditional St. John's Wort (SJW) oil macerate, and silver sulfadiazine (SSD) in a rat burn model, TQ and SJW were applied topically and systemically, while SSD was applied topically. Epithelialization, inflammatory cell response, granulation tissue, vascularization, and fibrosis were evaluated. Topically applied TQ accelerated epithelialization in wounds and provided granulation, vascularization, and fibrosis effects. Topically and systemically applied TQ improved vitamin E levels, but reduced total antioxidant status (TAS) and 8-hydroxy-deoxyguanosine (8-OHdG) levels. Topical SJW decreased granulation and vascularization, while topical and systemic SJW reduced total oxidant status (TOS), malondialdehyde (MDA), and 8-OHdG levels. However, it increased TAS and vitamin E levels. Topical SSD decreased TOS, 8-OHdG, and MDA levels. Topical and systemic TQ strengthened antioxidant defenses, decreased oxidative damage, and sped up the wound healing process. In particular, SJW oil increased epithelialization in topical application, but it was less effective in systemic use. Although SSD reduced oxidative stress, it was found to be less effective in promoting wound healing, and delayed granulation and fibrosis. Thymoquinone showed significant protective and healing activity, while SJW was found to be locally effective, but less effective systemically, and it was suggested that SSD should be used with caution and that it should be used in combination with antioxidants to reduce its potential negative effects on wound healing [66].

As observed in the studies mentioned above, ointments prepared from medicinal plant mixtures were found to be effective in comparative studies with silver sulfadiazine due to their effective compounds. Although there is no similar study regarding all the plants or ointment formulation used in our study, our study was planned considering that the plants may be found effective compared to SS due to the similar phytochemicals (polyphenolic and terpenic metabolites) they contain. In our study, within the context of formulation development, *Vitis vinifera* L. seed oil, *Trigonella foenum-graecum* L. seed oil, and the *Olea europaea* L. oil macerate of *Helichrysum italicum* (Roth) G. flowers and *Matricaria recutita* L. flowers were used as raw materials, and they were blended with Cera alba (beeswax) and white petrolatum for the preparation of ointments. The formulations were subjected to rheological property and texture profile analysis to assess their physical and mechanical properties, enabling the optimization of composition ratios. Following formulation optimization, *in vitro* wound healing (scratch assay) and cytotoxicity assays were conducted to evaluate the safety and efficacy of our distinct ointment formulations. Ultimately, the optimized formulations underwent further testing using an *in vivo* burn wound model in mice [67-70].

## 2 MATERIALS AND METHODS

### 2.1 Materials

Standardized *Vitis vinifera* L. seed oil, *Trigonella foenum-graecum* L. seed oil, and the *Olea europaea* L. oil macerate of *Helichrysum italicum* (Roth) G. flowers and *Matricaria recutita* L. flowers were purchased from FitoBio, Istanbul, Türkiye. Cera alba and petrolatum were purchased from Merck KGaA, Darmstadt, Germany. All other analytical materials were obtained from Merck KGaA, Darmstadt, Germany.

### 2.2 Preparation of Ointments

In this study, pre-formulation evaluations led to the selection of two formulations containing botanical raw materials. The formulations (Table **1**) were prepared by hot-melt blending of an ointment base with phyto-active oils. Specifically, Cera alba (beeswax) and white petrolatum were combined *via* hot melting at 50°C in a steam cup within a water bath, with continuous stirring to achieve homogeneity. As the mixture's temperature decreased to 40°C, macerates and/or oils were added, followed by further stirring to ensure uniformity, thus meeting the structural and consistency requirements of the ointment.

### 2.3 Organoleptic Controls

Quality control assessments for colour, odour, appearance, and sensation were conducted on the prepared ointment formulations.

### 2.4 Rheological and Mechanical Properties

#### 2.4.1 Viscosity

**Fig. (1):**
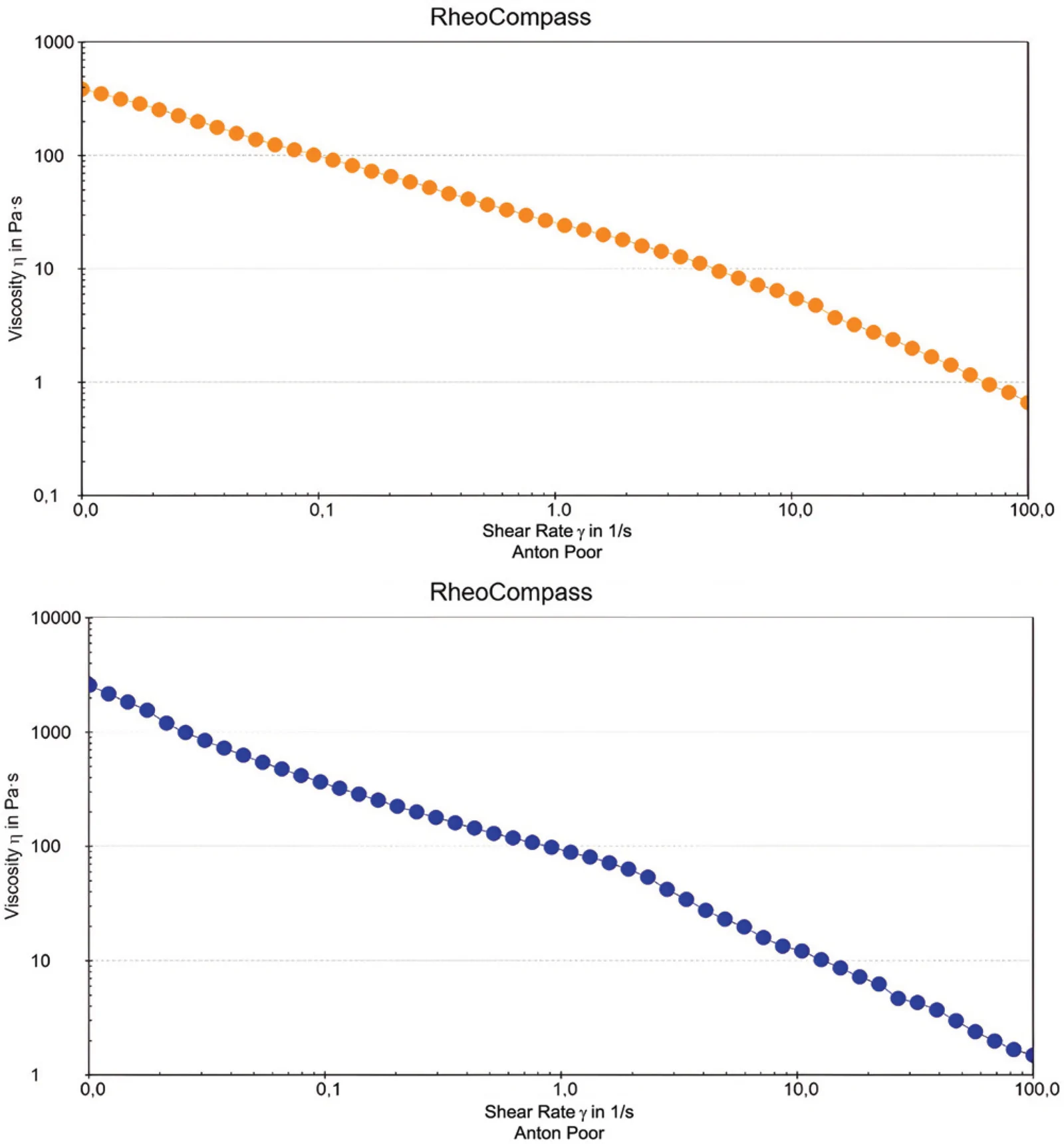
Flow rheograms of formulations at 32 °C obtained with a 50 mm diameter parallel plate. The tests were performed across a shear rate range from 0.01 to 100 s−1, with a total of 50 data points. **Note:** Orange: F1, blue: F2.

The flow behaviour of the prepared gel samples was evaluated by monitoring their viscosities through oscillation tests across a range of shear rates using a rheometer (Anton Paar MCR 102). Viscosity measurements were conducted using a 50 mm diameter parallel plate at a constant temperature of 32°C, maintaining a 1 mm gap between the plates. A 0.15 ml volume of each prepared solution was placed on the measuring plate, and the viscosity was then recorded. The tests were performed across a shear rate range from 0.01 to 100 s^−1^, with a total of 50 data points collected for each sample, allowing a comprehensive analysis of the ointments' viscosity and flow characteristics under variable shear conditions (Fig. **1**).

#### 2.4.2 Texture Analysis

The spreadability of the ointment formulations was assessed by using a texture analyser (TA-XT Plus, TTC Spreadability Rig HDP/SR) according to the back extrusion method and the device conditions (Table **2**) at room temperature, as presented in the literature [71-75]. The results have been presented in terms of firmness and work of shear, as shown in Fig. (**2**). The experiments have been performed in triplicate. Data collection and calculation have been performed using Exponent Connect Software, version 8.0.8.0.

### 2.5 Scratch Assay

Samples of F1 and F2 formulations were studied in the scratch assay using the HaCaT human keratinocyte cell line [CLS (Cell Lines Service) cat. no.: 300493] according to the method stated by Gökçe *et al*. [69]. For the scratch test, P18 HaCaT cells were seeded in 24-well culture dishes at a concentration of 5x10^5^ cells/mL and stored at 37°C in a medium containing DMEM-high glucose, 10% FBS (fetal bovine serum), 1% L-glutamine, and 0.1% penicillin/streptomycin. The cells were cultured in a humidified incubator with 5% CO_2_. After the cells covered the entire surface, the wound was created by scratching them along the midline of the well with a 200 µL pipette tip. Then, the medium was withdrawn, and fresh medium was added. Since the samples were creamy in nature, they could not be applied directly to the cells. A direct application would have interrupted the interaction of the cells with the medium. Thus, the formulations were applied to the cells in an apparatus called a PET membrane insert with a 0.4 µm pore diameter. In this way, the content of the product was ensured to mix with the compartment containing the cells upon contact with the nutrient medium. Different doses were applied to the cells. F1 and F2 formulations were applied to the cells in the initial study (400 µL). F1 formulation was applied in different doses (100 µL and 200 µL) in addition to a 400 µL application. Scratch distance was measured using the ImageJ program, and statistical analysis was performed using the GraphPad Prism 8 program. The findings obtained are shown in Figs. (**3****-****5**). Fig. (**5**) shows the change in scratch distance at different time periods.

### 2.6 *In vivo* Wound Healing Assay

*In vivo* studies were performed according to the Animals (Scientific Procedures) Act 1986 Amendment Regulations (SI 2012/3039) for the use of animals (supplied with certification from Kobay DHL A.S.); this research study adhered to internationally accepted standards for animal research, following the 3Rs principle, complying with ARRIVE guidelines, and it was approved by Kobay DHL A.S. local ethics committee with approval no. 658 (date: 10.02.2023).

The CD-1 male mice (24-30 g) aged 8-10 weeks were divided into 3 groups, with 6 mice in each group:

a) Untreated healthy group

b) Untreated burn group (control)

c) Treatment group (formulation F1 applied)

In the untreated healthy group, no wound model was created. The untreated burn group consisted of animals subjected to the burn model without receiving any treatment. The mice were anesthetized using ketamine/xylazine (60/8 mg/kg, IM) before the experiments were performed. The backs of anesthetized mice were shaved, and cylindrical moulds with a base area of 3.14 cm^2^ were created. Water with a temperature of 95°C was poured into the mould, which was then placed on the animals' backs with the upper and lower parts open for 15 seconds, and the wounds were closed with sterile sponges.

Once a day, 1 g of F1 ointment was topically applied to the burned area of the treatment group for 7 days. Tissue conditions were photographed every day for 7 days, and on the 8^th^ day, the tissues of the burned area and the surrounding connective tissues were separated and then frozen in liquid nitrogen for histological examination [69].

### 2.7 Histological Examination

The tissue samples of all groups were histologically examined according to the literature [69, 76]. The images obtained from the tissue sections were analyzed using a computerized video camera-based image analysis method (UTHSC Image software) by employing a high-resolution camera connected to the light microscope [Aver TV Studio Video Capture {version 4.21.0.0 (software) Aver Media Technologies, Inc.}].

### 2.8 Statistical Analysis

All data have been expressed as mean ± SD. ANOVA, followed by Tukey’s multiple comparison test, was performed using GraphPad Prism (GraphPad software, version 5.0 for Mac OS X, San Diego, CA, USA). The value of p < 0.05 was considered significant.

## 3 RESULTS AND DISCUSSION

### 3.1 Phytochemical Screening of Essential Oils

As a result of preformulation studies, two optimized formulations were selected and their organoleptic properties were examined. The ointments were all homogeneous and had soft sensations. Both formulations had green colour and herbaceous aromatic odour (Tables **1** and **3**).

The viscosity of ointments plays a crucial role in their formulation, stability, and therapeutic efficacy, particularly in wound care. It directly influences the ease of application, uniformity of distribution, and the rate of release from the base, which are critical for promoting wound healing and maintaining a moist environment. High-viscosity ointments provide better adherence to the wound site, enhancing localized delivery and forming a protective barrier. Conversely, lower-viscosity formulations may improve spreadability and patient comfort, especially on sensitive or irregular surfaces.

Both F1 and F2 formulations exhibited pseudoplastic non-Newtonian flow characteristics, and the viscosity values decreased as the shear rate increased. As can be seen in Fig. (**1**), F2 formulation presented higher viscosity than the F1 formulation. In the F1 formulation, the amount of Cera alba was 2% and that of white petrolatum was 34%; F2 formulation included these ingredients at the concentrations of 1% and 51%, respectively. The increase in the white petrolatum concentration led to an increase in the initial viscosity, as expected; F1: 382.98 Pa.s and F2: 2562.3 Pa.s.

The mechanical properties are among the important parameters in the performance of semi-solid formulations in the application area. In terms of mechanical properties, parameters examined include the ease of removing the ointment from its packaging and its ability to stay in place for a long time without spreading [77]. The TTC spreadability was used to determine the work of shear parameter, while the compression test was used to measure the force of deformation of the samples. The spreadability, which refers to deformation under an external load, is a more dynamic property that has been shown to be related to rheology through a material’s yield stress, the minimum shear stress required to initiate flow. Especially, the spreadability is inversely proportional to flow [78-81].

It has been reported that the correlation between spreadability differences and *in vivo* performance may help establish reasonable acceptance limits during the evaluation of Q3 equivalence for topical products [82, 83]. The texture analysis results are given in Table **4** and represented in Fig. (**2**).

**Fig. (2):**
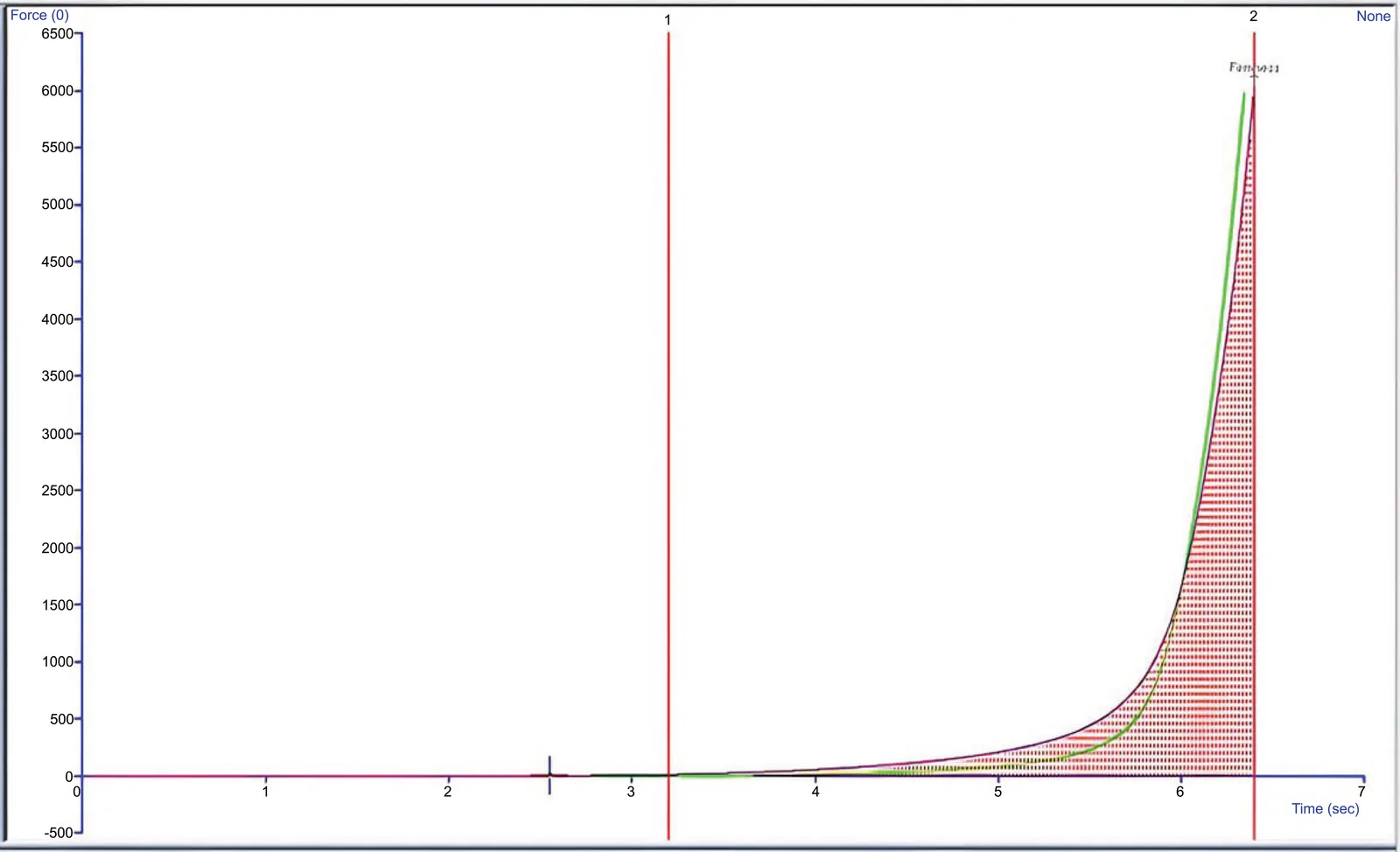
Photos of A. F1 and B. F2 (left to right on the bottom row) formulations. **Note:** C. Flow curves of herbal gel samples.

Both formulations created an easy-to-apply, soft, thin adhesive film layer. In terms of spreadability, they were found to be mechanically advantageous in terms of application to burn wounds (Fig. **2** and Table **4**).

### 3.2 Cell Culture Studies

The scratch assay was performed to examine the effects of ointments on wound healing *in vitro*. The HaCaT human keratinocyte cell line was used, and P18 HaCaT cells were seeded in 24-well culture dishes at a concentration of 5x10^5^ cells/mL and stored at 37°C in a medium cultured in a humidified incubator with 5% CO_2_.

**Fig. (3):**
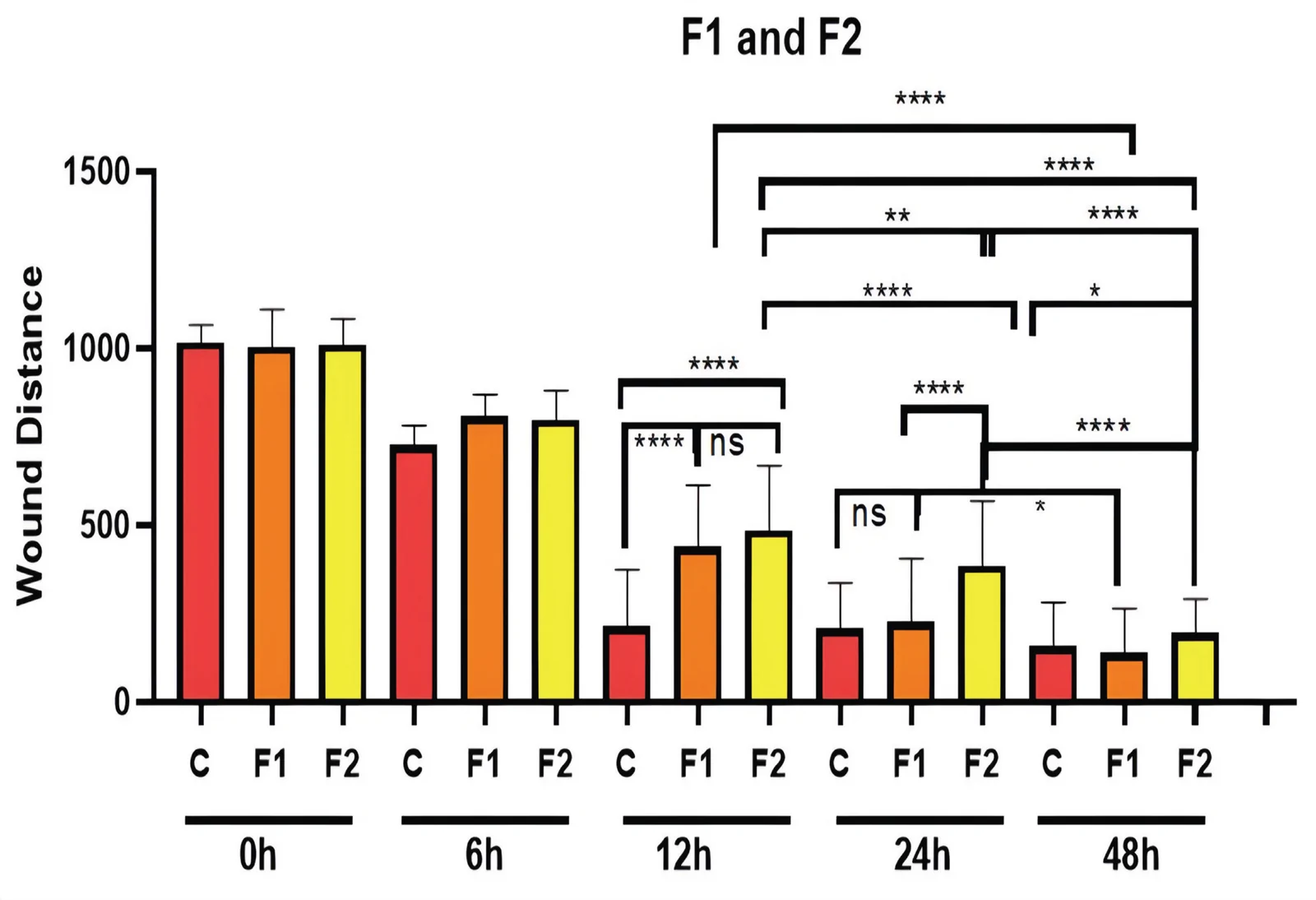
Change in wound distance for all groups over different time periods using the HaCaT human keratinocyte cell line. Measurements were taken at the beginning (0th hour), and at 6, 12, 24, and 48 hours. **Note:** C: control **** indicates *p*<0.0001. The bars show mean ± SD values.

**Fig. (4):**
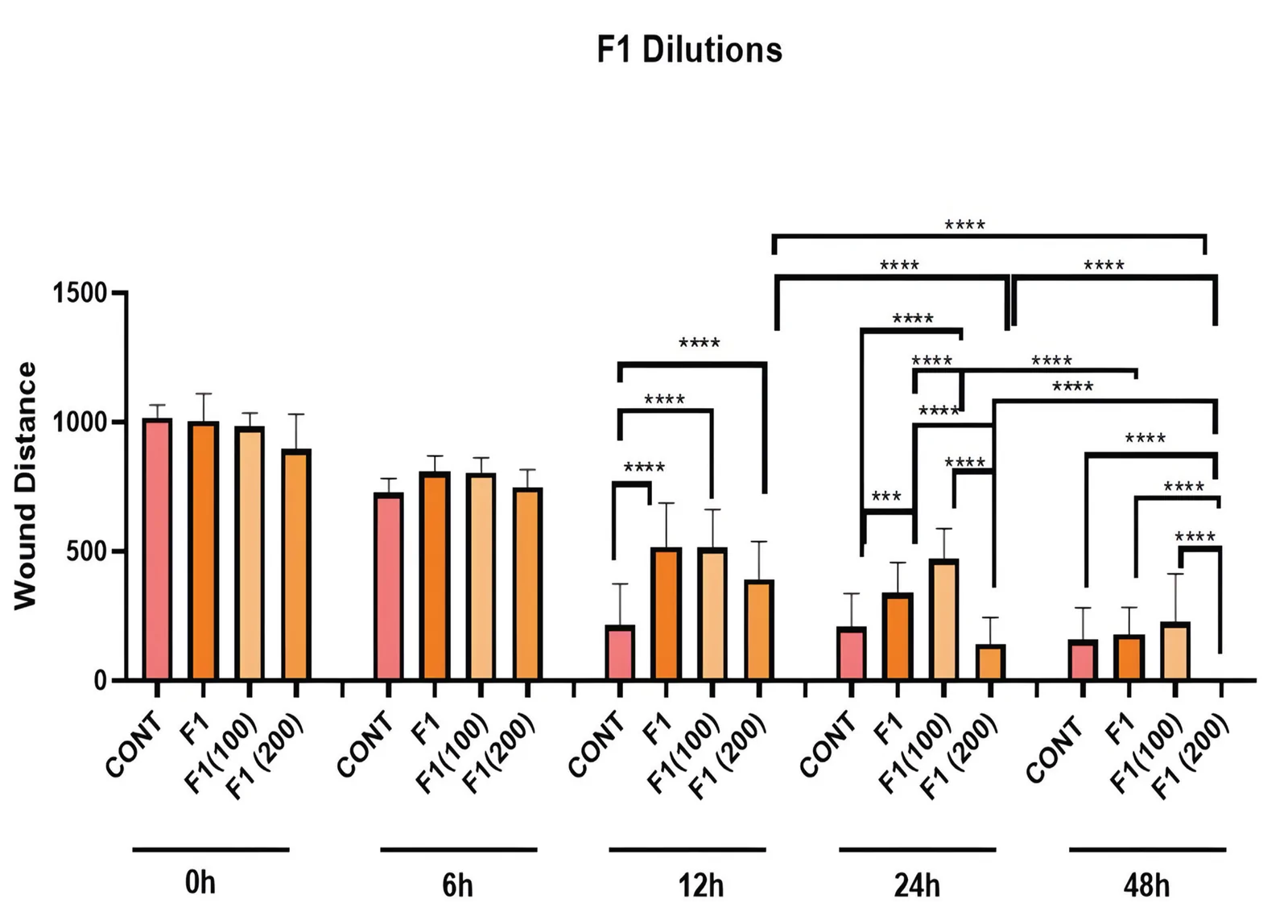
Wound distance changes induced by F1 formulation at different concentrations of 400 µL, 100 µL, and 200 µL for 48 hours using the HaCaT human keratinocyte cell line. Measurements were taken at the beginning (0th hour), and at 6, 12, 24, and 48 hours. **Note:** **** indicates *p*<0.0001. The bars show mean ± SD values.

**Fig. (5):**
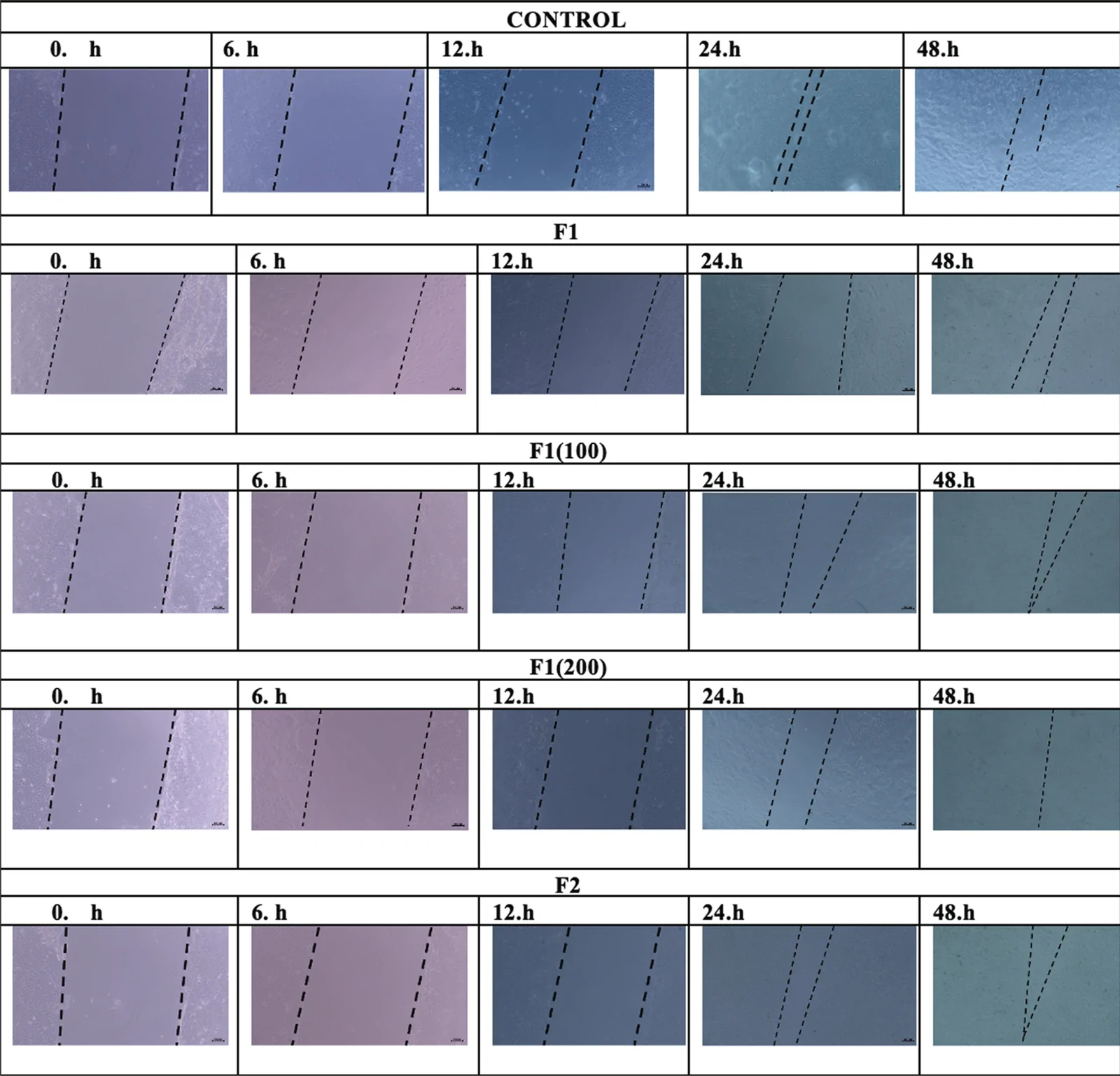
Wound distance closure extent and cell migration zone images obtained with phase-contrast light microscopy. Images were taken at 4X magnification. The scale bar was 100 µm. The control group included untreated cells.

Formulations F1 and F2 were applied at 400 µL per well in the initial cell study. As the contact time increased in the scratch tests, the differences between groups became more visible (Fig. **3**). In another study, for the first 24 hours, no wound healing could be seen in both groups for taurine-incorporated gels, and only traces of cell migration could be observed. The difference between the examined samples in terms of wound closure extent and cell migration zones could be seen at 72 hours [69]. In our study, at the end of the 24th hour, most of the closure occurred in the F1 formulation group. Since fast wound closure could be seen with F1 formulation, it was decided to observe the diluted concentrations and their effect on the wound closure *in vitro.* F1 formulation was applied in different doses of 100 µL and 200 µL, and the effect of dilution on cells was evaluated (Fig. **4**). In the group in which 200 µL F1 formulation was applied, the wound completely closed (100%) at the 48^th^ hour (Fig. **5**).

In this study, the formulations were developed using *Vitis vinifera* L. seed oil, *Trigonella foenum-graecum* L. seed oil, and the *Olea europaea* L. oil macerate of *Helichrysum italicum* (Roth) G. flowers and *Matricaria recutita* L. flowers. These materials were blended with Cera alba (beeswax) and white petrolatum for the preparation of ointments. Cera alba is known for wound healing properties; however, the higher wound closure effect was obvious with the use of plant materials in this study. The wound healing effect of the formulations was synergistic due to their phytoactives content. F1 formulation had higher phytoactives and cera alba and lower petrolatum in comparison to F2 formulation. The phyto-materials involved were antioxidants and anti-inflammatory agents. Thus, the synergistic effects could be attributed to these properties. One important finding of this study was the effect of the concentration of the F1 formulation applied. In *in vitro* studies, a critical concentration was identified at which the highest wound closure occurred. The same formulation with the highest and the lowest dose failed to close the wound. However, the F1 formulation applied at a 200 µL concentration managed to close the wound totally. This result was also confirmed in *in vivo* studies.

### 3.3 *In vivo* Wound Healing Evaluation and Histopathology Examination

**Fig. (6):**
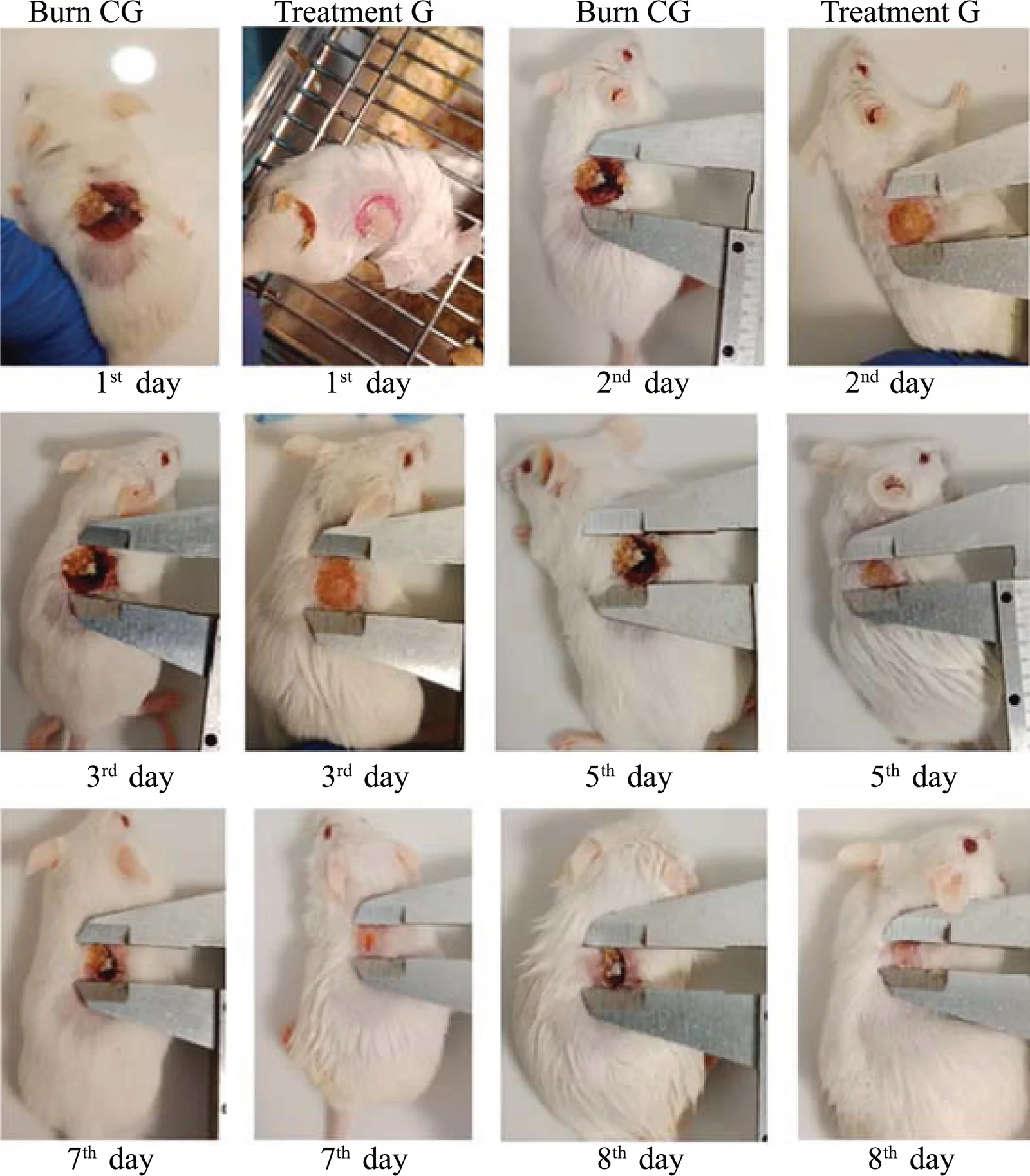
The skin images of the mice from the 1^st^ day to the end of the 8^th^ day according to *in vivo* experiments.

The wound healing test was conducted using the F1 formulation, and the treatment group showed successful outcomes, including lighter scarring, accelerated scab removal, flexible scar tissue, and a light-colored healing tissue appearance compared to the burn control group. It was observed that the group treated with the F1 formulation healed within one week without excessive crusting. This effect was believed to result from the osmoregulatory properties of the ointment applied to the wound area (Fig. **6**).

**Fig. (7):**
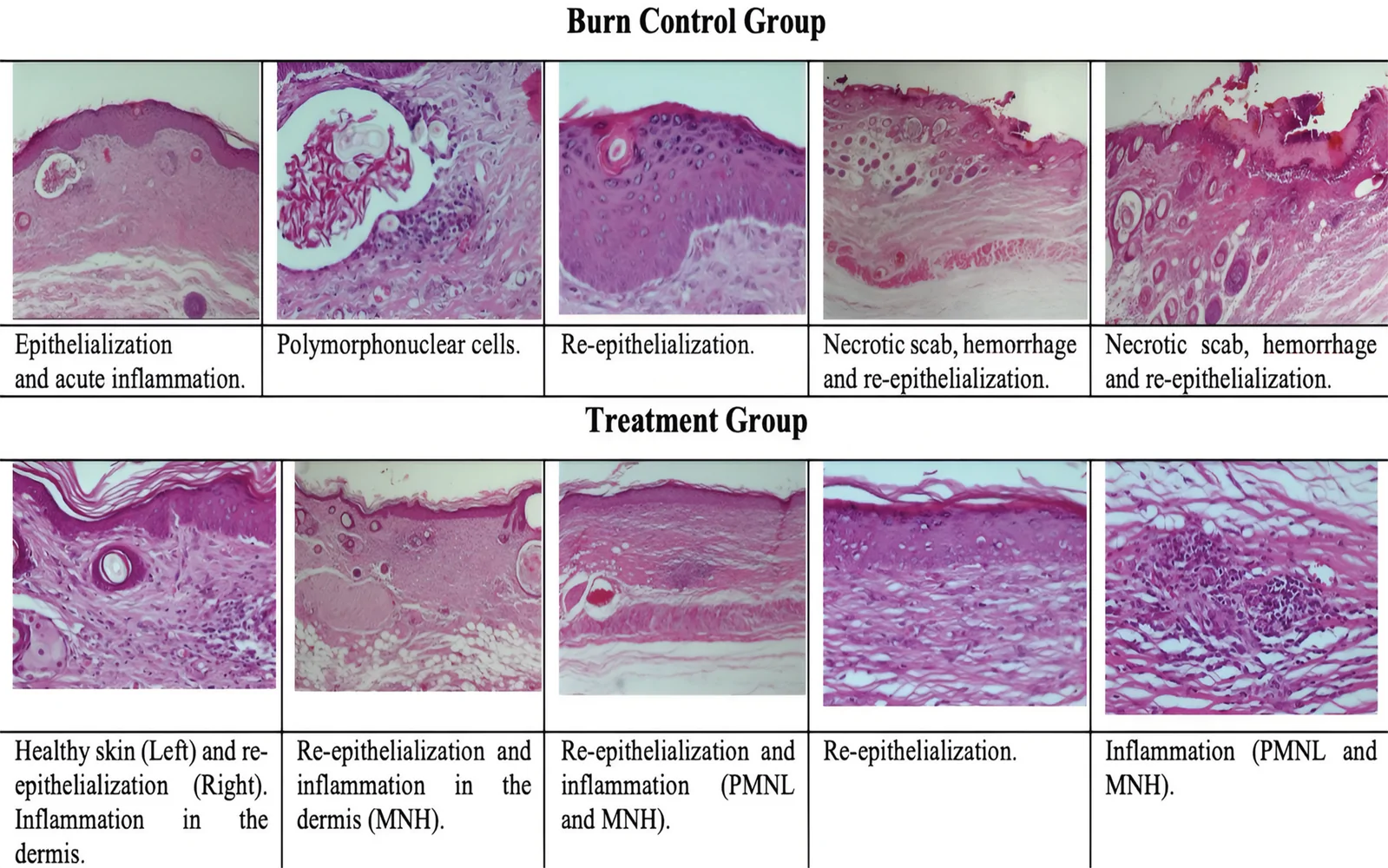
The light microscope images of skin tissue of the burned control group and skin tissue of the treatment group (J1-2). H&E x10.

In the burned but untreated control group, acute inflammation markers and polymorphonuclear cells could be determined. Necrotic tissue, haemorrhage, and re-epithelialization could be observed in the burned but untreated control group. However, in the treatment group, healthy skin formation and re-epithelialization could be seen with less inflammation in the dermis (Fig. **7**).

## CONCLUSION

This study demonstrated that the optimized phytochemical-based ointment (F1), containing the *Olea europaea* L. oil macerates of *Helichrysum italicum* (Roth) G. and *Matricaria recutita* L. flowers and the seed oils of *Vitis vinifera* L. and *Trigonella foenum-graecum* L., exhibited enhanced wound healing properties in both *in vitro* scratch assays and *in vivo* burn models. The formulation provided complete wound closure within 48 hours at a 200 µL dose *in vitro* and accelerated re-epithelialization with reduced inflammation *in vivo*.

The rheological and texture analysis confirmed the F1 formulation as suitable for topical application, forming a soft, thin, and adhesive film beneficial for wound healing. The findings indicated that the synergistic effects of the phytochemicals contributed to faster wound closure, reduced inflammation, and improved skin regeneration.

However, while the study confirmed the formulation’s efficacy, mechanistic insights into the biological pathways involved were not explored. Future studies should investigate the molecular mechanisms of action (*e.g*., inflammatory modulation, collagen synthesis, fibroblast migration) and compare the formulation with standard wound healing treatments to establish its clinical relevance.

## STUDY LIMITATIONS

This study is limited by the use of herbal ingredients containing complex phytochemical mixtures rather than isolated active compounds. While such formulations may exhibit synergistic effects, they complicate the elucidation of precise mechanisms of action. Additionally, determining the release profiles of active phytocomponents from formulations comprising multiple complex herbal ingredients presents another challenge, limiting the ability to fully characterize their pharmacokinetics and bioavailability.

## Figures and Tables

**Table 1:** The ingredients of F1 and F2 formulations (% content).

**Ingredients %**	**F1**	**F2**	**F1**	**F2**
*Helichrysum italicum* (Roth) G. macerate	16	12	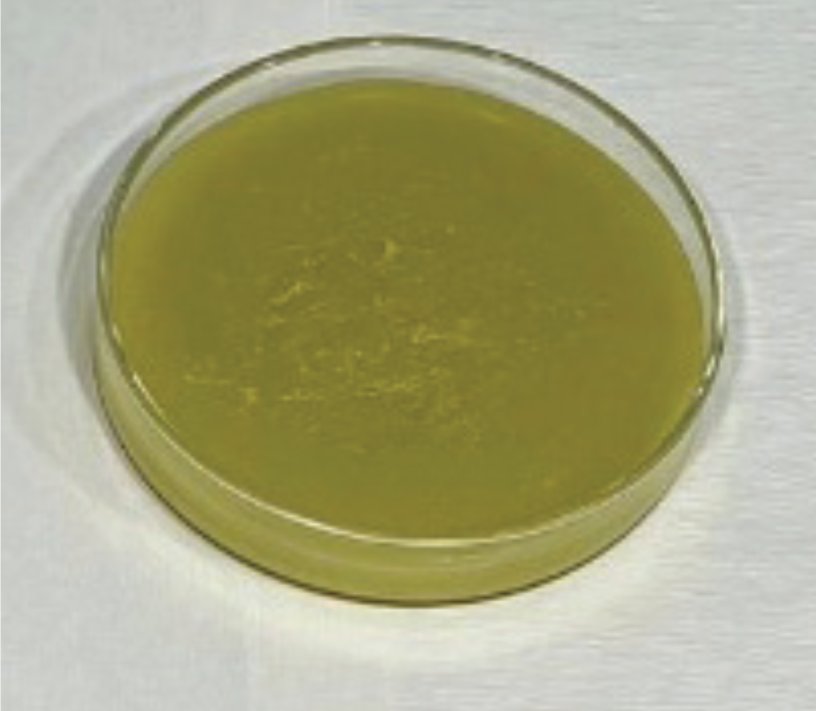	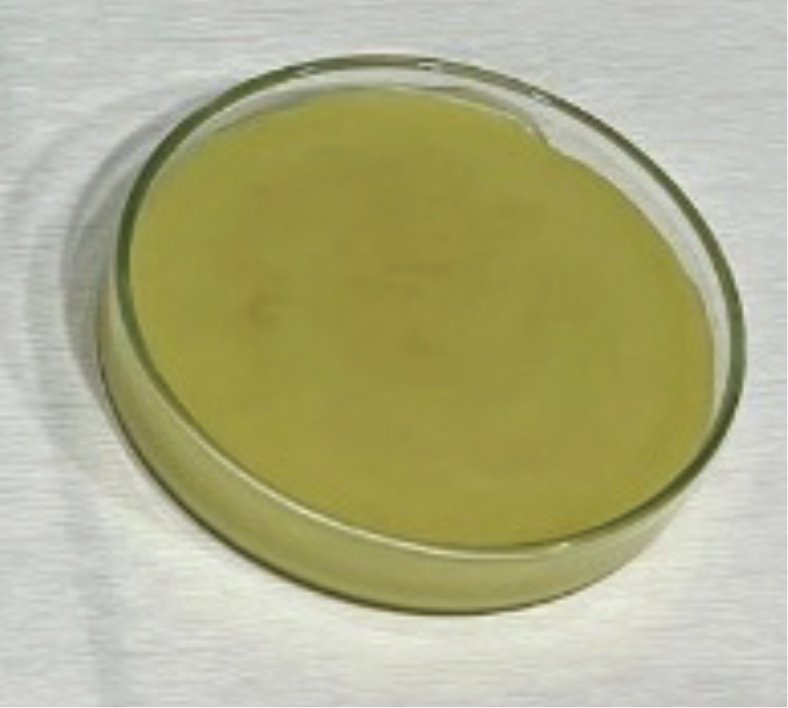
*Matricaria recutita* L. macerate	16	12		
*Vitis vinifera* L. oil	16	12		
*Trigonella foenum-graecum* L. oil	16	12		
Cera alba	2	1		
White petrolatum	34	51		

**Table 2:** Parameters set on the texture analyser using the back extrusion method.

**Parameter**	**Set Value**
Test mode	Compression
Option	Return to start
Pre-test speed	2.00 mm/s
Test speed	2.00 mm/s
Post-test speed	10.00 mm/s
Target mode	Distance
Distance	10.00 mm
Trigger type	Auto (force)
Trigger force	5.0 g
Break mode	Off

**Table 3:** Organoleptic properties of F1-F2 ointments.

**Characteristic**	**F1**	**F2**
Colour	Green colour	Light green colour
Odour	Pungent herbaceous, aromatic fragrance	Herbaceous, aromatic fragrance
Appearance	Homogeneous	Homogeneous
Sensation	Soft spreadable	Soft spreadable

**Table 4:** Mechanical properties of formulations.

**Formulation**	**Firmness (g)**	**Work of Shear (g.sec)**
F1	6183.7	3862.6
F2	6145.9	3945.3

## Data Availability

All data generated or analyzed during this study are included in this published article.

## LIST OF ABBREVIATIONS

FBS: Fetal Bovine Serum
HaCaT: Human Keratinocyte Cell Line
